# Can depth-based restrictions avoid seabird bycatch while maintaining catch in the Icelandic lumpfish fishery? A reply to Kennedy & Sigurðsson (2024)

**DOI:** 10.1098/rsos.240960

**Published:** 2024-07-24

**Authors:** Yann Rouxel, Hólmfríður Arnardóttir, Steffen Oppel

**Affiliations:** ^1^ BirdLife International Marine Programme, the Royal Society for the Protection of Birds Scotland, Glasgow, UK; ^2^ Fuglavernd BirdLife Iceland, Reykjavik, Iceland; ^3^ RSPB Centre for Conservation Science, Royal Society for the Protection of Birds, The Lodge, Sandy, UK

**Keywords:** bycatch mitigation, gillnet, depth restriction, lumpfish, guillemot, Marine Stewardship Council

Seabird bycatch in gillnets is an important conservation issue, with hundreds of thousands of seabirds inadvertently killed each year [1]. In an attempt to develop solutions to this problem, we recently tested whether a bird-scaring device could reduce seabird bycatch in the Icelandic lumpfish (*Cyclopterus lumpus*) fishery, which kills an estimated 8000 seabirds annually [2]. This investigation showed that the device was ineffective, but that seabird and marine mammal bycatch could be virtually eliminated if (i) lumpfish fisheries were restricted to waters greater than 50 m deep, and (ii) the patterns we found in our study area would apply to other fishing areas around Iceland [3]. We acknowledged that further research was necessary to explore whether the fish catch and bycatch patterns we found in our study area were representative of all of Iceland and that socio-feconomic considerations were necessary before any fishery restrictions could be imposed.

In a welcome contribution to work towards solutions for the bycatch problem in the Icelandic lumpfish fishery, Kennedy & Sigurðsson [4] now present novel data that shed further light on some of our assumptions. They confirm our findings that most seabird bycatch occurs in shallow waters less than 20 m deep, but that the bycatch of pursuit-diving seabirds (e.g. common guillemots, *Uria aalge*) occurs mostly in deeper waters. They further add data from another fishery (for Atlantic cod, *Gadus morhua*), which shows that some seabird bycatch even occurs in waters greater than 50 m deep, which complements the data obtained in our study focussed only on the lumpfish fishery [3]. Unfortunately, Kennedy & Sigurðsson [4] did not present data on marine mammal bycatch, which peaked at intermediate depths in our study [3] and should be considered when exploring solutions to reduce non-target bycatch in the Icelandic lumpfish fishery [5].

By using data from logbooks, Kennedy & Sigurðsson [4] show that very little of annual lumpfish landings come from depths greater than 50 m, which is largely a consequence of very little fishing effort occurring in waters greater than 50 m deep. Because the lumpfish fishery targets spawning females, seafloor habitat is critical to their distribution and productive fishing grounds have traditionally been in waters less than 50 m deep [4]. As our original contribution stated, a blanket ban on fishing in waters of less than 50 m around Iceland is therefore neither desirable nor practical and may have socio-economic consequences that need to be explored [3].

We propose that in areas where existing data suggest that fishing in waters greater than 50 m deep is both practical and beneficial (e.g. zones D, E and F), such regulations could be trialled to contribute to a reduction of bycatch in the Icelandic lumpfish fishery. According to the Marine and Freshwater Research Institute [6] nearly half of the fishery’s total seabird bycatch occurs in those three zones, which includes nearly 40% of all black guillemots *Cepphus grylle* killed in this fishery. Trialling depth-based restrictions in zones D, E and F could therefore help reduce bycatch levels of an endangered species in Iceland and provide data about fish catch and economic consequences for fishers that could be used to inform bycatch reduction measures elsewhere.

In other areas, data sharing and collaborative research might facilitate the development of alternative solutions. For example, using detailed fish catch records could improve species distribution models of lumpfish and identify potential fishing areas that have not been traditionally exploited [7–9]. Similarly, the experimental deployment of alternative fishing gears and experimental closures in both time and space could potentially reveal how fish catch could be maintained while simultaneously reducing seabird and marine mammal bycatch. Research to support alternative solutions is urgently needed, particularly in areas with high bycatch rates where depth-based measures might not be appropriate. For instance, area B represents 40% of the total marine mammal bycatch, including 75% of the grey seal *Halichoerus grypus* bycatch (listed as Vulnerable in Iceland), and a third of the total seabird bycatch in this fishery (including 40% of all black guillemot bycatch) [6].

We agree with Kennedy & Sigurðsson [4] that solutions to the seabird bycatch issue in the Icelandic lumpfish fishery need to be tailored to the specificities of each fishing zone. We call for the trialling of depth-based restrictions where possible, and for the development of alternative mitigation solutions elsewhere. Identifying sustainable solutions will require active collaboration between the fishing industry, research institutes and conservationists to ensure that the Icelandic lumpfish fishery can maintain its Marine Stewardship Council certification [10]. We welcome future collaboration efforts with Icelandic stakeholders to permanently reduce bycatch in this fishery and ensure a sustainable coexistence of the fishing industry and marine megafauna around Iceland.

## Data Availability

This article has no additional data.
